# Our Christmas Supplement

**Published:** 1905-12-09

**Authors:** 


### OUR CHRISTMAS SUPPLEMENT.

### Good Cheer for Hospital Supporters.

### TWENTY YEARS OF SOLID ADVANCEMENT.

This journal celebrates its twentieth Christmas Day this year. Our aim at the outset was to make hospitals live in the eyes of the world. We determined to vastly increase the knowledge of every class of public and benevolent institution, and this increase of knowledge has corrected not a few erroneous impressions. At the outset, we insisted, that the cause of hospitals was one to stir the blood, and awaken the purest, noblest, and most generous impulses of which men are capable. The work of hospitals calls into life some of the very highest and divinest forms of self-sacrificing duty. There are women at work within their walls, and men too, whose very presence in the world brings sweetness and purity along with it. If busy and idle men and women would but, now and again, spare a little time to acquire some intelligent knowledge about hospitals, their workers and their work, they could not but be both instructed and thankful, to know that such workers and such work are in their midst, and are the all-productive fruit of what are, sometimes, the mere remnants and fragments of a too-abundant wealth. Since these words were written nearly twenty years ago, the King's Hospital Fund and the League of Mercy have won to the cause of the hospitals many thousands of people, who to-day will endorse, out of actual experience, their accuracy and truth. As the outcome of all that has happened, and of the actual work done, during these twenty years, it is gratifying to realise public opinion now supports the view, that our great voluntary hospitals are, not only worthy of the utmost support, but also of unquestioning confidence.

A Living Force for British People.

Experience has taught us, that those who give most largely and generously to hospitals, and who are in the habit of preserving the special Christmas number of The Hospital for reference throughout the year, prefer that we should give them a clear statement of facts and found our appeals for the hospitals and charities upon that. The sentiment awakened by the hospitals is a living force in the hearts of English men and women. It needs no awakening by fine writing or eloquence. The overwhelming majority of British opinion, nowadays, is in favour of the permanent retention of the voluntary system of hospital support. All that is necessary to secure this end, is, to produce facts which cannot be disputed, to prove that the voluntary hospitals are doing all that is required of them, to adequately meet the needs of the humbler people, in the best and most practical way. The King's Hospital Fund has become the accredited centre from which this incontrovertible evidence is forthcoming with increasing force every year. The supporters of hospitals have been placed, by the work of the King's Fund, in a position to judge fairly, what are the respective merits and needs of each Metropolitan hospital, and they are also afforded an indication of the direction in which it is desirable to bring public opinion to bear, in order to remedy existing evils, or to stiffen the weaker brethren to attain to a higher efficiency in their management.

Unable to Part with their Money.

A gentleman, who is probably the most generous supporter of hospitals in this country to-day, and who has given enormous sums towards their maintenance, was lamenting, that in practice, it has proved to be impossible for some of the very wealthiest of people, to part with any considerable portion of their wealth in support of the suffering poor, or in other works of charity, during their lifetime. It is no use arguing the point, the fact remains that this type of wealthy person does exist amongst us, and that they are to be pitied for their lack of power to part with their money, wisely and well, during their lifetime, for they are thus deprived of realising the supremest happiness which wealth can bestow upon man or woman.

"The Best and Greatest Thing"!

Our friend then stated that the establishment of the King's Fund, and what it had done and was effecting for the hospitals in the Metropolis of the Empire, were amongst the best and greatest things which had been accomplished for England during the whole of its history. We believe this statement to be largely true, and that is why we have chosen the title of "Good Cheer for Hospital Supporters." Let us consider what have been the methods of the King's Hospital Fund, what it has accomplished, and what it is doing? From the outset, the King's Fund has taken infinite pains to work cordially and quietly, with the managers of the metropolitan hospitals. It has succeeded in ascertaining, fully, the inner working, the strength and weakness, the good and bad points, and the needs and merits of every institution to which the Fund has given a grant. It has instituted inquiries and independent investigations into moot points of administration, *i.e.* the relations between the medical schools and the hospitals, the existing arrangements for protection against fire, the full development and efficiency of the system of accounts upon a uniform basis, the ordinary expenditure and cost of various hospitals, and the publication of a complete and approved balance sheet by each institution. These are only a few of several steps in advance which have been taken on the initiative of the King's Hospital Fund. During the eight years of its working, this Fund has necessarily had brought to its notice various suggestions, to remedy existing defects, and to improve the management of individual hospitals. Such matters the Council have privately brought to the notice of the authorities concerned, especially in connection with expenditure, where, as in some cases, it still appears to be excessive. Every year this latter tendency is, however, being checked, more and more, so that every contributor may feel encouraged to continue his gifts.

### What has been Accomplished in London.

The King's Hospital Fund has accomplished many things of importance. Not the least of them is the organisation of a system of inspection of every hospital each year by medical men of wide experience, and laymen who take special interest in hospital management. As the Distribution Committee claim, these visitations, while they have admirably answered their immediate object by supplying reliable information regarding the merits and needs of the various institutions, have proved of very high value in stimulating improvements, both in construction and in all departments of administration. Defects have been pointed out, and their remedy has been powerfully promoted, by making the grants from the Fund conditional upon the carrying out of the measures that have been recommended. The suggestions, made by the visitors, have been almost invariably received, in a most friendly spirit, and new interest has been often aroused in those more immediately concerned in the several hospitals, leading them to increase their own subscriptions, or to promote, in other ways, by their individual exertions, the needed improvements. Work of this kind, well and carefully carried out as it has been done through the King's Fund, has immeasurably strengthened the voluntary hospitals, whilst the published remarks on the working of each hospital, and the report of the Distribution Committee, should afford every intelligent supporter, precise information, of the highest value, for his guidance.

### Bases of Gifts.

The King's Fund by its bases of awards has rendered very substantial service to the giving public by indicating the various directions in which money can be properly given to particular institutions. These bases include, (1) the opening of closed beds, (2) the wiping out or diminution of debt, (3) the carrying out of improvements by building or otherwise, and (4) the supply of various special needs and increased aids to general maintenance. As to (1) it may be pointed out that the King's Fund has given to Londoners the equivalent of a great free hospital containing 443 beds, that being the number of closed beds opened and made free to the poor, by the Fund. There is a growing feeling, which was shared by the late Charles Spurgeon, that any supporter of hospitals, who desires to give with intelligence, should seldom or never contribute anything to pay off a deficit or debt, incurred by the managers. Experience proves, that where the managers habitually permit annual deficits or debts to accrue, there the management is often slipshod and inefficient. It is now admitted, that no hospital, which is intelligently managed by capable men of business, need be in debt at all, because the managers always have it in their power to restrict their work to an expenditure, which is within the amount placed at their disposal by the public. It is satisfactory to note, too, that the larger givers, at any rate, are against any hospital or other charity committing themselves to large expenditure, upon buildings or upon maintenance, until they are in a position to know, that the funds required to defray its cost will be forthcoming, as and when wanted. The King's Fund has steadily set its face against wholesale deficits and excessive cost. In this way it is educating the giving public to realise, that the greatest reserves, at the disposal of the managers, lie in their power to restrict the amount of relief they give, to the absolute needs, of those only, who are actually entitled to receive free medical treatment.

### Good Work Done all the Time.

The King's Fund is doing good work too all the time. It has a system whereby the in- and out-patient departments, their actual condition and working and everything which appertains to them may be systematically examined year by year. The preparation of the returns and statistics published in the reports, the special needs of particular institutions, the condition of the fabric, structurally, hygienically and from a modern hospital standpoint, are regularly brought under review each year. So the work of the King's Fund has tended to improve the general efficiency of the better managed hospitals, whilst it withholds grants from any institution, the condition of which is found to be unsatisfactory. Without such a system, the most thrifty hospitals, which by careful management are kept free from debt, might receive less grants than their due, whilst those which are unthrifty and which accumulate large debts might get the bulk of the money. As it is, mistakes of this kind are rendered practically impossible, for every grant is made upon the actual ascertained position of each hospital, at the time it is awarded. The King's Fund has striven to abolish the objectionable practice of compelling in-patients to provide themselves with certain articles of food, including tea, sugar and butter, in addition to changes of personal linen and washing. One or two of the large hospitals, to their discredit, persist in continuing this objectionable practice, which properly had its origin in the days when hospitals were unhealthy, filthy and objectionable. The King's Fund, too, has worked steadily to bring about the amalgamation of certain of the smaller hospitals, including the orthopædic hospitals and those for diseases of the ear, throat and nose. There can be no doubt that one modern up-to-date and efficient orthopædic hospital, and one ear, throat and nose hospital of the same high efficiency, each with its country branch, would tend more to the general benefit of the patients, than any number of struggling institutions in London in rivalry with one another.

### Givers be of Good Cheer.

But we have said more than enough to convince the most sceptical that to-day every contributor to the voluntary hospitals of London may be of good cheer. As the Prince of Wales claimed a year ago, the King's Fund has shown its subscribers, that it is endeavouring, as far as possible, to assist the hospitals to spend their funds economically, and to husband their resources. The Fund is further willing to help them, if they so desire, to settle any vexed question, that may arise, with regard to defects of administration, whether internal or external, or on matters concerning reconstruction, removal or amalgamation. Such is the Prince of Wales's claim, and it proves H.R.H.'s real grasp of the most pressing hospital questions. Every supporter of the voluntary hospitals, then, may thank God and take courage. He is now afforded a guarantee that, with the hearty co-operation of the hospital managers, great changes are being promoted in many directions, which cannot fail to increase the popularity and efficiency of the London Hospitals. Thus everyone of intelligence is justified in contributing liberally to their support. Each hospital and institution is afforded an opportunity, to state its own needs and position in the pages which follow.

### Hospitals with 150 Beds and Upwards.

### GREAT ORMOND STREET CHILDREN'S HOSPITAL.

This hospital, the pioneer of children's hospitals in our Empire, is greatly in need of funds to carry on the good work done there; the extent of which proclaims itself in the statement that in fifty-three years nearly 1,000,000 children have been treated within its walls. The annual expenditure is £19,000 while only £12,000 can be relied upon. Moreover, a debt of £17,000 has been incurred in providing a nurses' home and a garden for the children. The latter is an essential agent in the building up of a child's weakened constitution after a long illness. No one who has once seen a sick poor child in its squalid miserable home could fail to be moved with the deepest pity for such a lot, and a subsequent visit to the same child in the hospital-ward where, even if suffering, it is almost happy in bright surroundings, cherished and cared for, would sufficiently illustrate the earnest need for help and all that it brings to thousands of suffering children. Secretary, Mr. Stewart Johnson.

### GREAT NORTHERN CENTRAL HOSPITAL.

The financial position of this hospital is a source of much anxiety to those responsible for its management. The neighbourhood of Islington, in which the hospital is situated, is suffering acutely from an unparalleled wave of commercial depression, and this is reflected in the decreased support which the inhabitants give to their local hospital. In every direction subscriptions and donations are falling off for the reason that people cannot afford the money. At the same time, the demands upon the hospital are greater than ever, and room cannot be found for many cases which apply. Last year 1,982 in-patients were received and 27,742 out-patients were relieved. At the present moment there is £6,000 due to the bankers and nothing is in hand to meet the Christmas bills of about £2,500 which will shortly become due. The funds of this hospital are most carefully and economically administered; it does an enormous amount of good work in a poor district, and well deserves a larger measure of support. Secretary, Mr. L. H. Glenton Kerr, Holloway Road, N.

### GUY'S HOSPITAL.

This well-known charity which extends so beneficial an influence over numbers of sick poor people who abound in South London, is in immediate need of funds. The hospital's settled income is £15,000 below its ordinary requirements; furthermore, out of the £100,000 appealed for last March to defray the cost of completing the building and renovations, £53,377 16s. has been received, leaving, however, a substantial deficiency to be supplied in order to free the institution from pressing liabilities. Among many necessary improvements a new department has been opened recently for the treatment of diseases by means of various rays—$x$-rays and Finsen light emanations—from which treatment such happy results are constantly obtained. The hospital contains 602 beds, but if the possibility of relief is to be extended to the utmost, an increase in public support is absolutely essential, and herein rests a grave responsibility with those who are able to help their fellow-men. At the Annual General Court of Governors of this hospital which was recently held, it was unanimously resolved "That the treasurer be requested to bring to the widest possible public notice the foregoing facts in the hope that their publication would secure at an early date the further support necessary to the prosperity and well-being of Thomas Guy's great Foundation."

### HOSPITAL FOR CONSUMPTION, BROMPTON.

There are now 418 patients in this hospital and sanatorium. Last year 1,771 in-patients and 12,391 out-patients were benefited by its efforts. The sanatorium, which is situated in a healthy part of Surrey, is found to be a most valuable acquisition to the hospital as it is there that the open-air treatment is applied to suitable cases. To efficiently maintain the hospital and home £35,000 is needed annually. If this sum is to be realised, however, a large additional income is required. For the public good as well as their own sake, it is desirable that the sick poor suffering from consumption should not be left in their own homes to spread the terrible disease. Moreover an institution of this kind affords the medical faculty such opportunities as tend to hasten the great strides which are being made towards the cure of the malady. Secretary, Mr. Frederick Wood.

### KING'S COLLEGE HOSPITAL.

After due consideration the committee have recently decided to move this hospital to a district of London more largely populated and where there is no similar institution in the vicinity. A site has been obtained, and it is hoped that the erection of the new building will shortly be in process Towards this undertaking nearly half of the £300,000 asked for has been subscribed, but there is still £150,000 needed. Work is still carried on at the present hospital, and the beds are always in demand, but there is a decrease in the out-patient attendances, due to the changes in the neighbourhood, which have been brought about by street improvements; and this proves the wisdom of placing the new hospital where it will be more needed and have a wider scope for its endeavours. The committee feel much anxiety regarding the present financial position, and appeal to the public for help in order to carry out this necessary and useful scheme and to continue the work of relief in the meantime. Secretary, Captain H. S. Tunnard, Lincoln's Inn Fields, W.C.

### LONDON LOCK HOSPITAL AND RESCUE HOME.

The Hospitals and Rescue Home accommodate 207 patients. During the past year 354 in-patients and 26,441 out-patients have been relieved. Patients who desire to lead a good life are cared for in the Rescue Home for a year, after which they are found situations. This shows the physical and social importance of the work carried on by these institutions. The demands on the charity are pressing, and as there are only 81 other beds in English hospitals devoted to similar work, it is most imperative that the operations of this Hospital and Rescue Home should not be curtailed. The Governors earnestly appeal for an increased number of annual subscriptions. Secretary, Mr. A. W. Cruickshank; offices, Harrow Road, W.

### MOUNT VERNON HOSPITAL FOR CONSUMPTION AND DISEASES OF THE CHEST.

Since 1840 this institution has grown from a small hospital to a great national charity. It now consists of a hospital at Hampstead containing 140 beds, a branch hospital at Northwood, and a largely attended out-patient department in Fitzroy Street. The cost of maintaining both hospitals and the out-patient department under the strictest possible economy cannot be less than £20,000 yearly. The institution owes a debt of £15,000 to its bankers, £5,000 of which has been promised if the balance is shortly raised. To clear this debt and to enable the charity to meet the demands upon it the Committee very earnestly appeal for the sum of £100,000.. The poor classes suffering from consumption have a special claim to help, for their ill-nourished condition and dark, over-crowded homes hasten and spread the terrible disease. There are 100 beds at the hospital closed for want of funds, while 300 patients are on the books in dire necessity of speedy relief. But they must stand outside closed gates—so appropriately called "the gates of hope"—hopeless, pathetic, suffering, until those with whom the power lies whisper the open sesame. Secretary, Mr. W. J. Morton.

### NATIONAL HOSPITAL FOR THE PARALYSED AND EPILEPTIC.

As the nature of the diseases of the nervous system makes it undesirable for many general hospitals and homes to undertake the responsibility of patients suffering from them—and the treatment calling for constant attention and observation as it does, making it almost impossible for such patients to be nursed efficiently in their own homes—a grave public need is supplied by this hospital. Last year 6,574 out-, and 1,113 in-patients were benefited by its treatment, but if the good work is to be carried on it must meet with more public support. In these days of hurry and strain, the special character and difficulty of the work done by this institution should appeal to all classes. The convalescent home is partly closed for want of funds. An increased number of annual donations is greatly needed to meet current expenses and to remove a yearly deficit of about £3,000. Secretary, Mr. G. Hamilton, Queen Square, W.C.

### ROYAL FREE HOSPITAL, GRAY'S INN ROAD.

Situated in a poor busy part of London, this hospital gives relief to large numbers of the poor sick people in the neighbourhood, for whom it is quite free, no subscribers' letters being required, and no charge being made to patients. Last year 2,364 in-patients and 42,050 out-patients and maternity cases were benefited by its efforts. The electrical and maternity departments add considerably to the annual expenditure, but this is justified by the excellent results which are obtained. During the past year it has been necessary to make some structural improvements in the wards, towards which King Edward's Hospital Fund has shown sympathy by making a special grant of £1,500 and H.M. the Queen by contributing £50. The improvements are now complete, and have cost £10,000. The demands upon the charity increase yearly, but if the good work is to be carried on with efficiency an increased number of subscribers is necessary. Secretary, Mr. Conrad W. Thies.

### SEAMAN'S HOSPITAL, GREENWICH.

The Seaman's Hospital Society comprises two hospitals and two dispensaries for the relief of sick and injured seamen of every nation. During the past year it has afforded relief to 26,000 patients. Its peculiar need is apparent in that a large number of these men are far from home and friends and often cannot speak the English language, to their embarrassment and distress in sickness. Moreover, their life of hardship, discipline, and peril is in itself an appeal for special sympathy and kindness in quiet days of illness. The growing demands on the charity necessitate extensions in the out-patient department at Greenwich, but this cannot be undertaken until the institution receives increased public support. The Committee earnestly solicit help for this necessary work and also for funds to carry on the charity. Secretary, Mr. P. Michelli, "Dreadnought" Hospital, Greenwich, S.E.

### ST. BARTHOLOMEW'S HOSPITAL.

Though the income arising from the endowments of St. Bartholomew's Hospital is adequate, with economy, to defray the annual cost of its maintenance, the Governors have no funds available for the work of reconstruction which has become urgently necessary. The first block of the new buildings has been commenced and is now well on in course of erection. It has cost about £125,000, towards which over £100,000 has been raised. The Special Appeal Committee are very anxious to maintain the tradition of the hospital "not to get into debt," and they ask the charitable public to help them by providing at once the money required for the completion of this first block. The block comprises an out-patients' and casualty department, with special facilities for the treatment of ophthalmic, skin, aural, throat, and dental cases, diseases of women, and of patients requiring electrical treatment. Accommodation is also provided for the resident medical and surgical staff. Clerk, Mr. Thomas Hayes.

### ST. GEORGE'S HOSPITAL.

Seeing that this hospital is situated in the wealthiest district in London, and that its commanding position must secure for it the daily observation of those who are well able to support it, no difficulty should be experienced in obtaining the necessary funds to enable it to meet the demands which are made upon it by the sick poor. Nevertheless, the accounts show that while the annual cost of maintaining the hospital amounts to £39,000, the income is only £27,000, the deficit being over £12,000. This lamentable condition should only require to be known to be immediately removed. Last year 4,530 patients were admitted to the hospital and 36,631 were treated as out-patients. The latter figure includes 429 women who were attended in confinement at their own houses. Contributions are also earnestly invited to the Samaritan Fund, which is of the greatest benefit to necessitous patients in sending them to convalescent homes, helping to support their families, and giving them assistance on leaving the hospital. Secretary, Mr. W. H. Davenport.

### ST. LUKE'S HOSPITAL FOR MENTAL DISEASES.

This old-established charity, founded in 1750 for the treatment of mental diseases on hospital methods, continues to do good work, and last year 284 patients were under treatment. The proportion of recoveries to admission was 52 per cent. Out of the 284 patients 247 paid less than the cost of maintenance, so this institution is doing good work, and especially to the "middle class" of limited means, whose claims in illness are too often forgotten. We are glad to learn that the Committee of Management are steadily working to effect the removal of the institution to a better site a few miles out of London, and that the difficulties in the way are gradually being overcome. A new feature is the private nursing staff to nurse mental cases at their own homes. Funds are urgently needed to help the hospital and its Convalescent Home. Secretary, Mr. W. H. Baird, Old Street, E.C.

### ST. MARY'S HOSPITAL, PADDINGTON.

This is the principal general hospital for the west and north-west districts of London. It is well known and fully appreciated by the sick poor who abound there, serving a population of upwards of half a million of whom it relieved last year nearly 50,000. But in spite of the wide and far-reaching help these numbers derive from it, the hospital does not receive the support it ought to do from the wealthy classes residing in and near its area. Annual subscriptions last year only amounted to £4,800, while the necessary annual expenditure was £31,500. The financial affairs of the hospital have now reached a serious crisis and if help is not speedily forthcoming, not only will it be impossible to open and furnish some of the new wards which have lately been erected but wards in the old hospital will have to be closed. This would be a grave curtailment of good, seeing that suitable patients are already being denied admission daily because there is no available accommodation. The sum of £60,000 is earnestly appealed for, £20,000 to defray the cost of finishing the building of the Clarence Memorial wing, £30,000 to furnish and properly equip this, and to make essential alterations in the old building, £10,000 to clear a debt on the general maintenance fund incurred largely through a falling off of legacies during the year. Secretary, Mr. Thomas Ryan.

### UNIVERSITY COLLEGE HOSPITAL.

University College Hospital is unique in hospital architecture, being built in the shape of a cross. It conveys to the passer-by that idea a cross ever must—of arms outstretched to gather in and temper the griefs and sickness of sorrowing humanity. And thousands of poor artisans seek and obtain there succour and relief. Since the new structure, which is almost entirely due to the late Sir J. Blundell Maple's generosity, was opened last September, the accommodation will enable the Committee to admit over 1,200 additional patients annually. This, however, will increase the cost of maintenance to £30,000 a year, which is a source of much anxiety to the Committee. No less than 2,744 in-patients were received in 1904, while 58,321 out-patients and casualties were treated, their attendances amounting to the large total of 204,124. The cost amounted to £23,557, but the reliable income was only £9,000, thus leaving £14,557 to be contributed by the public. Moreover, £8,000 stock had recently to be sold out, and donations to replace this amount are solicited. Secretary, Mr. Newton H. Nixon, Gower Street, W.C.

### WESTMINSTER HOSPITAL.

The Committee of this hospital are desirous of impressing its claims upon the well-to-do residents of Westminster. With rigid economy, it costs over £21,000 a year to maintain, towards which it is only able to raise £18,000, consequently there is a deficiency of £3,000. Last year it relieved 2,512 in-patients and 22,718 out-patients. Contributions will be gratefully received by the Secretary, Mr. Sidney Quennell.

### BIRMINGHAM GENERAL HOSPITAL.

The applicants during the past year have been so great that, in spite of the more chronic patients being sent to the Jaffray Branch Hospital and other convalescent institutions, there has been a constant list of patients waiting for admission. 5,496 in- and 61,362 out-patients have found relief during the past year. Last year there was a deficit in the annual income of £8,439. In a busy manufacturing town like Birmingham there is obvious need for a large efficient general hospital. Besides the ordinary population, the factory hands are numerous, notably poor, and their very occupation renders them liable to sickness and accident. If the increased work of the hospital is to be continued, more income is absolutely necessary, and the Board earnestly appeal for new subscriptions and donations. House Governor, Mr. Howard J. Collins.

### Hospitals with Under 150 Beds.

### THE CANCER HOSPITAL, BROMPTON ROAD.

This hospital, which is devoted to the treatment and alleviation of poor patients suffering from cancer, tumours, and kindred diseases, has a wide scope for its efforts. It has been the means of conferring great benefits upon those of the sick poor who have suffered from those maladies. Operations when indicated are performed by a medical staff possessed of the highest qualifications, and specially skilled in the treatment of cancer. The recent extension of the pathological department—which plays such an important part in the treatment of cancer—has made extra accommodation absolutely necessary, and the Committee have therefore decided to erect a nurses' home, the cost of which will be £5,000. To meet a deficiency in the hospital's income, £6,000 is needed, also £2,000 to pay for sanitary and hygienic alterations which the Committee, after careful consideration, have found it necessary to carry out. The efforts to alleviate so terrible a disease as cancer should meet with ready sympathy. Secretary, Mr. F. W. Howell, Fulham Road, S.W.

### EAST LONDON HOSPITAL FOR CHILDREN.

The fact that this hospital is situated in Shadwell leaves no room for doubt of its need. The very words "East London" indicate that its position is in the midst of poverty and suffering, a great deal of which falls on the shoulders of the many children who abound in that district. Some idea of the work this hospital is doing is found in the number of children to whom it has given relief. Last year there were 1,656 in-patients, as well as 397 at the seaside branch at Bognor, while 35,344 out-patients were treated. But the hospital does not receive the amount of support it ought to do owing doubtless to its situation. For this reason a double appeal is made, first for more funds to meet a heavy deficit, and secondly that those interested in the charity will visit the hospital, see for themselves how much it is needed, and make the institution more widely known. Secretary, Mr. W. M. Wilcox, Shadwell, E.

### HOSPITAL OF ST. JOHN AND ST. ELIZABETH.

Founded in 1856 and rebuilt in 1898, this hospital relieves that class of patients for whom the Metropolitan hospitals are not available. Patients are kept for any length of time when there is a prospect of doing them good, and as prolonged treatment and nursing is in nearly every case necessary before a complete cure can be effected, a great need is being supplied by this charity. All needing relief are received, irrespective of religion, and during the past year 271 in-patients were admitted. The Board rely upon continued and increased help from the public to carry on this commendable work. The annual income amounts to about £3,600, and the expenditure £4,000, so that a further £400 is required to enable this institution to pay its way. Secretary, Mr. C. Soggee, Grove End Road, St. John's Wood, N.W.

### LONDON HOMŒOPATHIC HOSPITAL.

This excellently appointed and ably conducted hospital was founded fifty years ago for the treatment of the sick poor according to the medical principle known as homœopathy. It was rebuilt in 1895, and the new hospital is held to be one of the best-arranged and most comfortable hospitals in the world. A few figures will illustrate its progress. In its first year its in and out-patients numbered 1,703; now 25,000 patients are relieved in the two departments. Notwithstanding the utmost economy in administration, it costs £10,000 a year to maintain the hospital, and help is needed to meet this heavy liability. Moreover, £4,500 is still owing to the Capital Funds for money withdrawn to pay for the new building. £2,100 of this is promised on condition that the remainder is forthcoming. Efforts are therefore being made which will enable the hospital to secure this liberal offer. Secretary, Mr. E. A. Attwood, Great Ormond Street, W.C.

### LONDON TEMPERANCE HOSPITAL.

On account of the ever-increasing number of out-patients, the present out-patient department has become quite inadequate. The only way to overcome the difficulty is to pull down the old building and erect one suitable to the pressing demands. This, means an expenditure of from £10,000 to £15,000, towards which King Edward's Hospital Fund has shown practical sympathy by making grants amounting to £2,400. The out-patient department is a potent factor for good, not only for those it relieves, but to the community at large, for so often prompt attention means subsequent economy by the prevention of greater and permanent disease. A glimpse at the out-patient department where the hard-working bread-winners and weary mothers gather, to obtain that relief which enables them to follow up their occupation, would serve to illustrate the great need for help, and the distress that would be caused, if relief had to be refused. Secretary, Mr. A. W. Bodger, Hampstead Road, N.W.

### METROPOLITAN HOSPITAL.

This institution is situated in one of the poorest and most crowded, and therefore most suitable, districts of London. It deserves a much larger measure of support than it has heretofore succeeded in attracting. Of its 111 beds 97 were daily occupied during last year, and it relieved 1,270 in-patients and 34,111 out-patients. During the last three years the average gross annual income has been £13,985, and the average gross annual expenditure £13,776. Some £20,000 are urgently required for a nurses' home in order to free for the reception of patients the wards now converted into cubicles. The secretary, Mr. Charles H. Byers, Kingsland Road, N.E., has for many years laboured assiduously and successfully to extend the resources of this important institution, which in the past has been far too little known to wealthy contributors to hospitals.

### NORTH-EASTERN HOSPITAL FOR CHILDREN.

Besides the ordinary expenditure this hospital has been seriously handicapped by additional expense, incurred through the necessity of accommodating the nurses in other premises and because the hospital does not possess a laundry. However, the Committee now feel able to undertake the building of a nurses' home and laundry, and it is most desirable that they should be completed as soon as possible, the Committee expecting to save thereby £900 yearly. To carry out the laundry scheme and make important alterations in the hospital heating system the sum of £4,100 is required. Moreover, £2,500 is needed immediately that the year may be closed free from debt as regards its maintenance fund. This year the number of beds in the hospital has been increased to 116, but so great are the demands for relief, that the patients in the wards exceed the ordinary number of beds. To enable this increased work to be carried on liberal support is necessary. Secretary, Mr. T. Glenton Kerr, Hackney Road, Bethnal Green, E.

### POPLAR HOSPITAL FOR ACCIDENTS.

Situated amongst a teeming population of poor hard-working people in a district which may be called the "workshop" as well as the "port" of London, this hospital is doing an excellent work. The demands on the institution have of late years greatly increased, and consequently further subscriptions and donations are very necessary. Secretary, Mr. Percy Rogers, B.A.

### ROYAL LONDON OPHTHALMIC HOSPITAL.

This institution, which was founded in 1804 and formerly known as the Moorfields Eye Hospital, is the oldest and largest eye hospital in the kingdom. It is well known and appreciated by thousands of poor people living in the thickly populated part of the East End, in which it is situated, and elsewhere in the Metropolis and country. There are 138 beds in the hospital and over 100 in-patients and 400 out-patients are relieved daily. The value of a free hospital of this kind can hardly be overestimated when it is remembered that even slight injury to the eye needs prompt and skilled attention if the sight is to be preserved, and this the poor often could not get if a fee were necessary for relief. Moreover, many kinds of work the poor are compelled to do for a livelihood puts a great strain on their sight and so renders them more liable to disease. This fact establishes a claim upon those in whose power it lies to aid the efforts that are made to preserve the eyesight of the poor working-classes and prevent the calamity of blindness. Secretary, Mr. Robert J. Bland, City Road, E.C.

### VICTORIA HOSPITAL FOR CHILDREN, CHELSEA, S.W.

This hospital has recently been enlarged in order to meet pressing demands and facilitate the rendering of skilled assistance to those needing it in the neighbourhood. The new building contains 104 beds, besides 50 cots at the Convalescent Home at Broadstairs, where 500 to 600 children are sent yearly to effect a complete recovery. To efficiently maintain the hospital and Home an additional sum of £3,000 in annual subscriptions is needed, and of the new building expenditure there is still a debt of £9,000 to be paid. Sick children, even more than adults, require, in addition to medical attendance, skilful nursing and trained observation, and since the foundation of the hospital in 1866 33,819 in- and 128,109 out-patients have been provided with this necessity. As one glances back over the years and mentally sees this army of little sufferers with un-childlike upturned faces, sick and wasted, appealing for pity, the thought of them being insufficiently nursed in their own miserable homes, with chance of recovery thereby lessened, and pain intensified tenfold, is not to be borne, but happily as long as the hospital is supported that need never be. Secretary, Mr. H. G. Evered.

### Hospitals with Under 100 Beds.

### CHELSEA HOSPITAL FOR WOMEN.

This hospital contains 50 beds, and was founded in 1871 for the treatment of poor women and gentlewomen in reduced circumstances. It fulfils a great need in being able to offer to its patients a degree of privacy unattainable in many larger institutions. The number of severe cases it treats is abnormally high, but notwithstanding this fact, the results of their treatment are exceptionally good. The Convalescent Home, with 22 beds, at St. Leonards-on-Sea, continues to prove, as was expected, an invaluable adjunct in the restoration to health and strength of large numbers of patients who have undergone severe operations at the hospital. The steps that are taken to keep abreast of the latest demands of medical science are most necessary for the attainment of the best results. At the same time they constitute an increasing charge on an already inadequate income, and donations and new annual subscriptions are therefore much needed, and will be gratefully received by Mr. Henry E. Wright (Hon. Treasurer), or the Secretary, Mr. Herbert H. Jennings, at the hospital, Fulham Road, S.W.

### HAMPSTEAD GENERAL HOSPITAL.

THE new building at Haverstock Hill, which Princess Christian has consented to open on December 16, comprises accommodation for 64 patients, and this number will be increased when funds permit the extension of the building. The sympathy which the King's and other funds have extended to the institution are marks of its need and efficiency, also the large number of in- and out-patients whom the hospital has relieved, as recorded in the report. Beds are provided for patients able to pay from 12s. a week towards their expenses, and these patients can be attended by their own medical men if desired. £8,000 is needed to remove a debt on the building fund, and if additional beds are to be maintained, an increase of income to the amount of £2,000 is necessary. Secretary, Mr. George Watts.

### HOSPITAL FOR WOMEN, SOHO SQUARE, W.

THE varied and complicated diseases which women are liable to, and do so frequently suffer from, enhances the value of a hospital especially designed to alleviate their pain and distress. This hospital is much appreciated by the patients for whom it is intended, and since its foundation in 1842, upwards of 207,000 have been relieved. The hospital contains sixty beds, all of which are constantly in demand, and for the maintenance, which costs over £6,000 a year, there is great need for increased annual donations. Secretary, Mr. David Cannon, Soho Square, W.

### NORTH-WEST LONDON HOSPITAL.

THIS hospital has a wide scope for its efforts, being much needed in that district of the Metropolis in which it is situated. The institution is quite inadequate to the demands of the North-West district, and the Committee feel the urgent necessity for erecting a modern hospital with increased accommodation. Last year 621 in-patients were treated, while there were 43,620 attendances of out-patients. Less than £700 is to be relied upon from annual subscriptions, while the yearly expenditure amounts to £4,500. Funds are greatly needed if the relief and succour the hospital affords is not to be curtailed, and unless help is speedily forthcoming the Committee will have the ordeal of facing a deficiency of £2,000 on January 1. Secretary, Mr. A. Craske, Kentish Town, N.W.

### QUEEN CHARLOTTE'S LYING-IN HOSPITAL.

THIS institution supplies poor women, irrespective of religion or nationality, with medical attendance in the hospital, or the attendance of a trained midwife in their own homes, as the case may require, during their confinement—a time when they need all possible care and comfort. All patients are invited to contribute whatever they are able towards their expenses, but such means are, of course, slight and the hospital's reliable income is only £2,000 against a necessary annual expenditure of £6,000. In spite of enlargement of the hospital and a new nurses' home having been erected in recent years, the demands on the charity have been so great that additional premises have had to be acquired to enlarge the nurses' home, the cost of which will exceed £10,000. It will therefore be seen that this hospital stands in much need of assistance. Donations may be sent to the Secretary, Mr. A. Watts, Marylebone Road, N.W.

### ST. JOHN'S HOSPITAL FOR DISEASES OF THE SKIN.

THIS is the only hospital in London or the neighbourhood especially built to meet the requirements of patients suffering from skin diseases, and as one of the greatest difficulties of dealing with these cases is the prolonged period they need for treatment, this makes them unsuitable patients for general hospitals, and enhances the value of a special hospital of this kind. The new hospital is now open, and has a department for *x*-ray, Finsen, and other electrical treatment. Much necessary expense has recently been incurred at the in-patient department in the suburbs for repairs, electric light installation, linoleum for the wards, and other improvements, which greatly add to its efficiency. There are ever-pressing demands on the charity for relief, and in order that this may not be misplaced, inquiries are made as to applicants' circumstances. The sum of £10,000 is needed for the new building in Leicester Square, and an additional £8,000 to purchase the freehold of the in-patient department in Uxbridge Road. Secretary, Mr. G. A. Arnaudin, Leicester Square, W.C.

### HOSPITALS WITH LESS THAN 50 BEDS.

### THE BELGRAVE HOSPITAL FOR CHILDREN.

This is the first hospital to move from the North to a densely populated district on the South side of the river, where the need of it has been fully proved by the great number of patients, many of whom have to be denied admission owing to insufficient income. Over £3,000 a year is required to keep two wards open, containing thirty cots, but the income is far short of this; moreover, there is a debt of over £8,000 on the building. The Secretary would most gladly answer, by personal call or letter, any questions about the work among the sick poor of South London, or the financial position of the hospital. This Institution should not be permitted to suffer by its removal from the neighbourhood of the wealthy to an essentially fitting position among the poor, and it is therefore confidently hoped that the increased support which it requires to carry on an increased work will be forthcoming. Secretary, Mr. F. Stuart, Clapham Road, S.W.

### CENTRAL LONDON OPHTHALMIC HOSPITAL.

This hospital, which has been established over sixty years, is quite unendowed, and owing to loss of many supporters by death is now in great need of funds. Last year 12,820 patients were relieved, 369 of which were in-patients, yet the cost of maintenance only amounted to £1,870 for the year. These figures prove the need of the hospital and the economical lines on which it is worked. The expiration of the lease falls shortly, and as the old building is out of date and quite unsuitable, it is most desirable that a new building should be erected. £6,500 has been subscribed already, but the Committee are unwilling to start until the whole necessary sum is forthcoming, and appeal for funds that this may shortly be possible. The efforts to preserve sight and prevent one of the most terrible afflictions—its loss—must commend themselves to all benevolent people. Secretary, Mr. H. R. S. Druce, Gray's Inn Road, W.C.

### CHILDREN'S HOSPITAL, PADDINGTON GREEN.

This hospital is supported entirely by voluntary contributions and is quite free to sick poor children. It contains 46 cots and in 1904 763 in-patients were relieved within its walls, while the out-patient attendances reached a total of 53,782. There is a convalescent home attached to the hospital at "Fair View," Slough, which accommodates 16 patients. In 1904, 188 were sent there, thus satisfactorily completing their cure and leaving free much-needed cots for other little sufferers. Towards a debt of £1,500 which is owing to the bankers the Committee have received £600 and a promise of £500 if the other £400 is obtained, for which they earnestly appeal. A visit to a children's hospital, besides making manifest the pathos of their suffering, proves how much can be done to lessen it. Treasurer, George Hanbury, Esq., 28 Prince's Gate, S.W.

### HOSPITAL AND HOME FOR INCURABLE CHILDREN, NORTH COURT, HAMPSTEAD, N.W.

It is a pretty safe conjecture that no two words in the English language paint instantaneously so sad a mental picture as "incurable children." Poor little weary souls weeping "ere the sorrow comes with years." A glimpse of their pinched, wan faces, often ill-nourished, and crooked little bodies, would readily awaken sympathy. To alleviate the sorrows of such the above institution was founded in 1875. This year the home was moved from Maida Vale to much more suitable and healthy quarters in Hampstead—an undertaking which has already proved beneficial to the general tone of the children's health. The new building has, of course, incurred extra expenditure, to meet which £5,350 is needed, exclusive of funds necessary for the carrying on of the institution. Honorary Secretary, Mr. Henry Sewell.

### HOSPITAL FOR GENTLEWOMEN.

The object of this Hospital is to afford medical attendance free, and a home and skilled nursing for the sisters and wives of professional men of slight means. The condition of the present house necessitates the removal of the hospital, and the cost of adapting the new house to hospital requirements, will mean an expenditure of £3,000, which sum the Committee look for help to raise. An increase in donations is also required to meet deficiency in the annual income. The embarrassment of this class of patient should be keenly apparent to their more fortunate sisters. Lady Superintendent, Miss Tidy, 90 Harley Street, W.

### ST. SAVIOUR'S HOSPITAL.

This hospital, which is quite unendowed, supplies home comforts, medical skill, and hospital advantages to ladies in reduced circumstances, who otherwise would be obliged to receive help from a charitable institution or inadequate nursing in their homes. An average of 100 patients are treated annually, but the small fees they are able to pay towards the necessary expenses leaves a large deficiency to be met by voluntary contributions. The pinch of poverty is hard for gentlewomen to face at any time, but more especially in sickness, when they are not so well able to cope with difficulties. The endeavours to help this class of patients should appeal strongly to all more fortunate women whose every need is supplied in their own homes. The Honorary Secretary will be glad to receive communications at Osnaburgh Street, N.W.

### SAMARITAN FREE HOSPITAL FOR WOMEN.

This hospital is quite free to all poor women for the treatment of diseases peculiar to the sex; poverty and sickness being the only qualifications necessary for admission. Funds are urgently needed for there is a debt of £7,000 to be recovered to pay the cost of the new out-patient department, improving the nurses' quarters, and to build a new theatre. In addition the hospital is dependent upon public support for £6,000 yearly towards its maintenance and for these sums the Committee earnestly appeal for increased help. Secretary, Mr. W. G. King, Marylebone Road, N.W.

### The Economic Value of Hospitals.

The associations which suggest themselves to the popular mind in connection with hospitals are rather personal and sentimental than social and economic. Individual experiences of cases in which suffering has been relieved, death averted, and health restored, appeal to sympathies that have an acute feeling for the patient immediately concerned and for those more or less directly related to him, and this personal position commands issues in comparison with which money values are necessarily thrust into a purely subordinate place. Thus it happens that the work of hospitals is for the most part regarded in its aspect of beneficence to the individual sufferer, and claims for the support of that work are apt, not unnaturally, to appeal principally to that sympathy with individual misfortune which makes the whole world kin. And certainly no one with any knowledge of what hospital opportunities mean to the individual sufferer will be disposed to suggest that this aspect of the work is presented in phrases which are in the least in advance of the facts. Further, apart from any question of sternly practical values, and beyond the help and succour of individuals, it will be allowed that hospitals play a not unimportant part in the community by keeping before the public mind a practical realisation of active benevolence which helps to quicken the social conscience to realise the truth that human society involves mutual obligations among the individuals of whom it is composed.

### Hospitals and the Working Capacity of Communities.

There exists, however, a wholly different basis upon which may be rested a secure justification of the existence of our hospitals, and from which they may be seen to be rendering to the community services and value that may be weighed in strictly practical scales, and may be subjected with confidence to the unsentimental criticism of a stern political economy. To recognise the position to which reference is now made, it is necessary to appreciate the effects produced by the work of the hospitals upon the working capacity of the community by the prevention and cure of disease, and to understand that influence and power in both of these directions have been much extended in recent years as a direct outcome of the hospital system. The most conspicuous example of successful prevention of disease is of course to be found in the checking of outbreaks of the specific fevers which in earlier days were accepted as so many necessary visitations, and each of which claimed as victims its list of young lives having a greater or less measure of promise of efficient service. Who shall pretend to estimate what economic value has been contributed to the community by men and women who, under conditions different from those of our modern day, would have been swept into an early grave by typhus or small-pox or some other epidemic influence? Take again the treatment of diphtheria by an antitoxin serum, and consider how by this agent alone lives have been saved for work and purpose which but a few years ago would most infallibly have been lost. It is obviously impossible to pretend to make any detailed statistical statement on these two illustrative cases, or, indeed, on the general truth which they represent, but it is certain and beyond dispute that the diminution of epidemic disease has meant an enormous economic gain to the community. Now the point which must be emphasised is that the knowledge by which this diminution has been rendered possible has in the main resulted from the exact and minute study of disease which can hardly be undertaken except when large numbers of patients are collected together in circumstances that render careful and scientific observation possible. It is thus facts are provided in sufficient numbers and so related in time as to render possible the appreciation of the true nature of the processes of disease, and out of this has grown the knowledge which has rendered control and prevention effective. Of course it may be urged that our general hospitals have now nothing to do with epidemic diseases in the ordinary sense of that term. That is quite true, but the present position by which the community is protected and numberless valuable lives are saved every year is a direct outcome of the study of these diseases in general hospitals and of the discoveries there made that only by isolation of all cases of these diseases could epidemics be prevented. In short, one great economic contribution made by the hospital system to the community has been a knowledge of the nature of epidemic diseases and of the means by which these diseases can be prevented or kept under control. That forms an item in the hospital claim which cannot be lightly disregarded.

### How Hospitals benefit each Individual.

Two further observations may be made on this point. First, it is right to emphasise the statement that not only the community as a whole, but every individual class, has been a sharer in these benefits. Hospital patients, cared for in the first instance as individual sufferers, have been the sources of knowledge in reference to the nature and prevention of disease from which has sprung protection and salvation for vast numbers of persons who are far removed from the necessity for the succour and aid of charity. In the second place, it is fair to claim that what has been accomplished in the past will find parallels in the present and future. Indeed, there can be no doubt that such an enormous economic question as the nature and cure of cancer will in all probability meet its solution as a direct consequence of knowledge resulting from the collection together of numerous victims of the disease in special wards or hospitals. Life is so short and the opportunities of the private individual are so limited that hospital experiences are almost a necessity to the recognition of some large truth capable of commanding a considerable department in the prophylaxis or treatment of disease. When, therefore, such a truth is discovered, and individuals of all rank and condition get the benefit of it, and when, at the same time, the community gains protection and its efficiency is increased by an added number of competent workers, it is right and proper that the hospital system should receive its fair share of credit and should be recognised as an actual and potential factor in the economic success of the entire community. Here, then, the claim is made that from the work of our hospitals has followed knowledge of enormous economic value in reference to the prevention and cure of disease, and similarly it is urged that experiences of the same order are likely to be repeated in the future.

### Increase to Earning Power of Workers.

The position is very much the same in considering the social and economic value of the individual patient. Out of hospitals broken and damaged men and women are returned in a restored condition again to take their part in the scheme of social organisation. But for the hospital many of these as workers would be permanently disabled, and would become charges upon local or national funds. Further, it is right to recognise that in this respect the hospitals, by modern methods of treatment—themselves the results of hospital experiences—are enabled considerably to shorten the average period of residence in the wards. Especially is this true of surgical cases. The practice of antiseptic surgery means every day the prompt restitution to active life of workers who in a former generation would have remained helpless in bed for weeks or months. Hence every hospital serves a larger constituency than was formerly possible, and has a more ample range of economic service.

### Some General Charities.

### ASSOCIATION FOR THE ORAL INSTRUCTION OF THE DEAF AND DUMB.

The propagation throughout the Kingdom and Colonies of an improved method for instructing deaf and dumb children is the work of this Association. The children are taught to speak as well as understand language, and when they are sufficiently advanced they receive a general education. Adults who have become deaf can also have instruction in lip-reading as a substitute for hearing. There is also a training school for teachers, but the small fees the pupils are able to pay is quite inadequate to meet the expense of maintaining the Training College and school, and the income is £600 annually short of expenditure. Director, Mr. William Van Praagh, 11 Fitzroy Square, W.

### THE BRITISH ORPHAN ASYLUM, SLOUGH.

This charity benefits orphans of the middle classes whose parents have been unable to leave provision for them. There are now 200 children in the schools, and every effort is made to give them a good education, that in after years they may regain the social footing their parents held. The reliable income is deficient by over £4,000, moreover, £7,550 is owing to the bankers, to clear which, and for funds to carry on the work, donations are earnestly solicited. There are not many institutions in aid of this class of afflicted children, and they should therefore find sympathetic supporters in the more fortunate members of their own class. Secretary, Mr. C. T. Hoskins, 27 Clement's Lane, E.C.

### CHILDREN'S HOME AND ORPHANAGE.

There are ten branches in connection with this Home, which exists to supply those who have lost their parents or are neglected and unprotected, with home life, influence, and education. Sick and afflicted children are also received, and cared for in the Homes. There is an Emigration Home for boys in Canada, from which 1,800 children have been sent out to situations. As far as numbers are concerned, 1,709 children have been aided in one year; but those who would gain a real knowledge of the deep and far-reaching benevolence of the institution should study the annual report. Children taken from degraded homes, slums, and even from the prison-gates, have been saved from miserable lives and been enabled to live useful and happy ones. It has been found desirable to enlarge the Birmingham and Frodsham branches, and for this expenditure and the cost of maintaining the institution funds are greatly needed. Communications should be sent to the Rev. Arthur E. Gregory, D.D., Bonner Road, N.E.

### THE CHRISTIAN COMMUNITY.

This society, of which the late Sir George Williams was President, devotes itself to teaching and helping the poorest of the London poor. It is difficult for many people to recognise their own moral starvation when all their efforts must be brought to bear on the prevention of physical starvation, and this Community tries to bring out the better nature of the destitute and lonely, while it recognises and assists their material needs. Its operations include weekly visits to 15,000 inmates of workhouses in a year, 27,708 children's dinners and breakfasts, and a country holiday for a week to 400 children and adults. The work is carried on by 450 voluntary workers. Donations will be gratefully accepted by the Secretary, Mr. Jas. Atkinson, The Hall, London Street, Bethnal Green, E.

### FREE CONVALESCENT HOMES.

Anyone visiting the wards of a hospital, and seeing the weary-stricken faces lying there, would realise the absolute necessity of a thorough change of air and scenes if health, after prolonged weeks of pain and suffering, is to be completely restored. Owing to the ever-pressing demands for beds, it is often impossible to retain a patient during the convalescent stage, and yet they are certainly not fit to go straight from ward comforts to their poor homes. Since 1840 the Metropolitan Institution has, by providing free convalescent homes, been making escape from this difficulty possible. It now comprises four homes containing a total of 550 beds, and receiving annually 7,000 patients. Last May a new home, containing accommodation for 71 men, was opened at Little Common, Bexhill. £13,000 is needed yearly to maintain these homes; nearly the entire amount has to be obtained from voluntary sources. In addition, there is a debt of £10,000 owing to defray the cost of completing the new home, and to free the institution from these burdens an increased number of donors and subscribers is needed. Secretary, Mr. Alex. Hayes, 32 Sackville Street, W.

### IRISH DISTRESSED LADIES FUND.

This Fund assists gentlewomen who, owing to the Irish Land Agitation, have not only been reduced in circumstances, but deprived of even necessary means of subsistence. Pensions are granted to those who through advanced years or infirmity cannot help themselves; others who can, are helped in their endeavour to find suitable work. In addition there are "Illness," "Educational" (for applicants' children), and special grants made in cases of urgent necessity. The receipts to September 30 were £1,373, but the expenditure during that period amounted to £1,579 10s. 4d., and the deficiency had again to be supplied from the reserve fund, which is now so small as to be insignificant. The Committee therefore urgently appeal for further support. Secretary, General W. M. Lees, 411 Oxford Street, W.

### LONDON ORPHAN ASYLUM.

From amongst the great number of orphans who exist in our Empire 6,644 have received benefit from this charity. There are 460 children at present in the school, and a great many seeking admission, whom the managers would like to admit in January if the necessary public support could be relied upon. The annual expenditure amounts to £13,200, and this sum is required from voluntary sources. In spite of the managers having sold stock to the value of £2,000 last January, there is still £2,500 owing to the bankers. To lovers of humanity the example and teaching of the supreme Lover of humanity has left no room for doubt that to the best of our ability it is a duty to succour, as well as pray for, "all those who are desolate and oppressed." Secretary, Mr. H. C. Armiger, 21 Great St. Helen's, E.C.

### LONDON SOCIETY FOR TEACHING THE BLIND.

Since its foundation in 1838 this institution has grown steadily; now it is a large commodious building situated in the North-West of London. The children are taught in the school—besides reading—ordinary English subjects and, when sufficiently advanced, some occupation such as typewriting, basket making, pianoforte tuning, etc. This not only enables them to earn a livelihood but, work being ever a great leveller of sorrows, they are helped to forget their affliction. Besides teaching and training children, blind journeymen are given work at the institution for which they are paid weekly wages. The charity is in great need of increased support, particularly in annual subscriptions, which last year only reached £523, while £1,000 is required. A visit should be paid to the institution to gain an idea of the real value of the work it is doing. Secretary, Mr. Thomas H. Martin, Swiss Cottage, N.W.

### MARY WARDELL CONVALESCENT HOME FOR SCARLET FEVER.

This Home, which was founded by Miss Wardell in 1884, has since been the means of benefiting 4,000 patients from various institutions and sources. Patients recovering from scarlet fever, if confined in their own homes, not only run a greater risk of falling victims to its many complications, but are a source of danger to others. Thus the Home supplies a grave need. The patients' fees are not nearly sufficient to maintain the Home, and a settled endowment fund is very much needed. Miss Wardell has devoted her interests for twenty-five years to the Home, and it is her greatest wish, and that of the Committee, to see such a fund established. Subscriptions and donations may be sent to Messrs. Barclay and Co., 1 Pall Mall East, S.W., or to Miss Wardell, Stanmore, Middlesex.

### ROYAL MATERNITY CHARITY OF LONDON.

This charity, which was founded in 1757, exists for the purpose of providing midwives and medical attendance to poor married women at their own homes free of charge. Over 3,000 such annually are attended at their confinement, and for ten or more days afterwards. Many poor women at this supreme time of their lives are unable to leave their home and duties to obtain hospital advantages. It is an anxious period, even in homes where every luxury is to be had, and in those where abject poverty prevails it is beset with danger, and calls for skilful aid, help, and sympathy. The amount of relief given has had to be reduced, through loss of income, and £1,000 is owing to the bankers. Capt. G. L. B. Killick, Secretary, 31 Finsbury Square, E.C.

### THE ROYAL ORPHANAGE, WOLVERHAMPTON.

This institution, which originated in 1850 as a temporary home to accommodate nineteen fatherless children, has grown to such an extent that there are now 320 children, gathered from all parts of the kingdom, in the orphanage receiving home comforts and education; though 400 could be received if funds permitted. During recent years the institution has lost many old friends and supporters—in the last twelve months over 600 guineas has been lost in annual subscriptions alone. Further support is urgently needed to make up this loss and prevent curtailment of so admirable a work. The influence of such an institution is a national good, for besides material succour it endeavours to supply a father's wisdom, discipline, and tenderness, so necessary in youth, if in after years these poor children are to be qualified to combat with the world's snares and difficulties. Secretary, Mr. W. Hamblett.

